# Factors associated with self-medication in users of drugstores and pharmacies in Peru: an analysis of the National Survey on User Satisfaction of Health Services, ENSUSALUD 2015

**DOI:** 10.12688/f1000research.17578.2

**Published:** 2020-01-24

**Authors:** Diego Urrunaga-Pastor, Vicente A. Benites-Zapata, Edward Mezones-Holguín

**Affiliations:** 1 Unidad de Investigación para la Generación y Síntesis de Evidencias en Salud, Universidad San Ignacio de Loyola, Lima, 15024, Peru; 2 Epi-Gnosis Solutions, Piura, Peru; 3 Facultad de Ciencias de la Salud, Universidad Peruana de Ciencias Aplicadas, Lima, 15023, Peru

**Keywords:** Adults, Pharmacies, Self-Medication, Universal Coverage, Insurance, Health Services Accessibility, Peru

## Abstract

**Background:** Irresponsible self-medication is a problem for health systems in developing countries. We aimed to estimate the frequency of self-medication and associated factors in users of drugstores and pharmacies in Peru.

**Methods:** We performed a secondary data analysis of the 2015 National Survey on User Satisfaction of Health Services (ENSUSALUD), a two-stage probabilistic sample of all regions of Peru. Non self-medication (NSM), responsible self-medication (RSM) and irresponsible self-medication (ISM) were defined as the outcome categories. Demographic, social, cultural and health system variables were included as covariates. We calculated relative prevalence ratios (RPR) with their 95% confidence intervals (95%CI) using crude and adjusted multinomial logistic regression models for complex samples with NSM as the referent category.

**Results:** 2582 participants were included. The average age was 41.4 years and the frequencies of NSM, RSM and ISM were 25.2%, 23.8% and 51.0%; respectively. The factors associated with RSM were male gender (RPR: 1.35; 95%CI: 1.06-1.72), being between 40 and 59 years old (RPR: 0.53; 95%IC: 0.39-0.72), being 60 or older (RPR: 0.39; 95%IC: 0.25-0.59), not having health insurance (RPR: 1.89; 95%CI: 1.31-2.71) and living in the Highlands region (RPR: 2.27; 95%CI: 1.23-4.21). The factors associated with ISM were male gender (RPR: 1.41; 95%CI: 1.16-1.72), being between 40 and 59 years old (RPR: 0.68; 95%IC: 0.53-0.88), being 60 or older (RPR: 0.65; 95%IC: 0.48-0.88) and not having health insurance (RPR: 2.03; 95%CI: 1.46-2.83).

**Conclusion:** Around half of the population practiced ISM, which was associated with demographic and health system factors. These outcomes are the preliminary evidence that could contribute to the development of health policies in Peru.

## Introduction

Self-medication is a practice that represents a public health problem worldwide ^1^, mainly in developing countries, where it is an important issue for the health systems. The World Health Organization (WHO) defines self-medication as the selection and use of medicines by individuals to treat self-recognized illness or symptoms ^2^. In addition, the concept of responsible self-medication (RSM) is based on the treatment of diseases and conditions using medicines that do not require a prescription for their sale, due to their safety and effectiveness when correctly used ^2^. For that reason, there are over the counter (OTC) medicines, which would respond to the concept of RSM ^3^. To study these behaviors is relevant in developing countries of Latin America.

The frequency of self-medication differs according to the country and context evaluated. Studies have reported self-medication prevalence ranging from 27% to 90.1%. In Asia, a study made in India reported a prevalence of 71% ^4^, while in Iran it was 35.4% ^5^. In Europe, research studies in Spain reported a prevalence between 14% and 90.1% ^6– 8^. In Latin America, Colombian studies presented prevalence ranges from 27.3% to 55.4% ^9– 11^, whereas in Brazil, it oscillated from 31% to 86.4% ^12, 13^. In Peru, a previous work found a self-medication prevalence of 56.7% in an urban area of Lima ^14^. Then, we consider relevant to research the prevalence of self-medication since it has benefits and risks ^15^.

Self-medication practice can entail suffering serious adverse effects. In addition, the concomitant use of several medicines may develop interactions that could increase those adverse effects ^1, 16^. Even OTC medicines used inappropriately and irresponsibly can represent a risk for the consumer ^1, 15, 17^. Among the main benefits of the RSM, we can mention the increased access to pharmaceutical products, the reduction of unnecessary medical appointments, and of the expenses in healthcare services by the government ^15^. For this reason it is important to evaluate the factors associated with the irresponsible self-medication practice (ISM) ^18^.

Among the main conditions associated with self-medication practice are demographic, social, cultural, personal and health system factors. Age, sex, socio-economic status and educational level are frequently related to self-medication practice ^10^. Among the personal factors associated with self-medication are having good results after self-medication, the belief of having experienced manageable similar symptoms previously, the fear of being diagnosed with a serious disease and the need to alleviate symptoms prior using healthcare services ^16, 19, 20^. Regarding healthcare system, it has been described that self-medication is related to the easy access to medicines in drugstores and pharmacies and the lack of access to healthcare services, which in turn, is associated with the lack of a health insurance ^19^. Thus, it is proved the multifactorial character of the self-medication practice.

In this context, despite its relevance, we have not found nationwide studies that had evaluate the frequency of self-medication in users of drugstores and pharmacies in Peru. The objective of this study was to estimate the frequency of RSM and ISM, and to identify the factors associated with such practice in a population-based sample.

## Methods

### Study design

We performed a secondary data analysis using the fourth questionnaire of the National Survey on User Satisfaction of Health Services (ENSUSALUD) of 2015. ENSUSALUD is an annual questionnaire, the first edition was carried out in 2014, and was applied to internal and external users of healthcare facilities. The survey has been executed by the National Institute of Statistics and Informatics (INEI, by its Spanish initials) in collaboration with the National Superintendency of Health (SUSALUD, by its Spanish initials) ^21^.

ENSUSALUD is composed by six questionnaires, the fourth questionnaire evaluated the drugstores and pharmacies users who were within the perimeter of two blocks around the 181 healthcare facilities that provided health services for the Ministry of Health and Regional Government (MINSA-GR, by its Spanish initials), Social Security System (EsSalud, by its Spanish initials), Health Service of the Armed Forces and Police (FF.AA.PP, by its Spanish initials) and Private Practice (CSP, by its Spanish initials) evaluated in the first and second questionnaires ^21^. The study was carried out in 179 drugstores and pharmacies in the 25 regions of Peru.

### Population, sample and sampling

**Figure 1.**
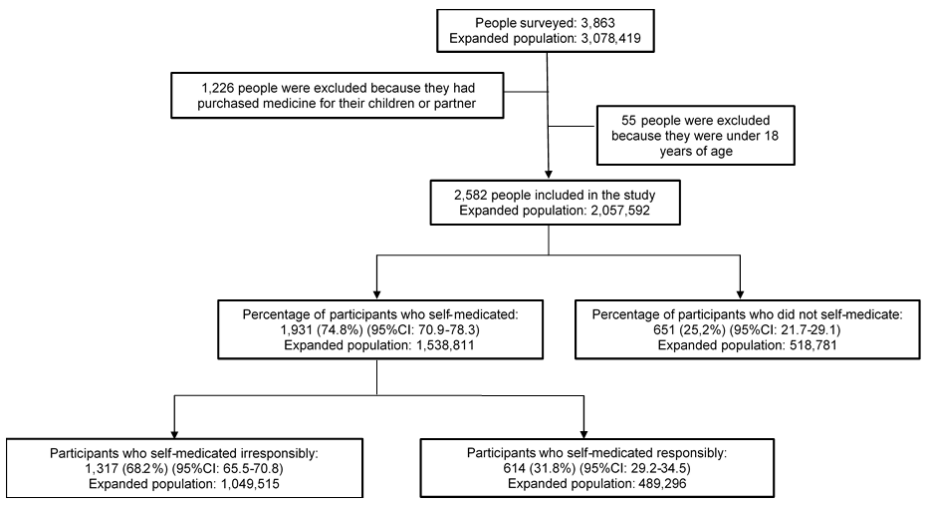
Flowchart of participants selection, ENSUSALUD 2015.

The population was composed of clients of drugstores and pharmacies, who were surveyed after the purchase of a medicine. In total, the fourth questionnaire included 3863 participants. The sample size calculated of 3,863 participants represented an expanded population of 3,078,419 people; participants were not excluded due to lack of data or incomplete records ( Figure 1). The calculation of the sample size used a design effect of 1.2 and a satisfaction possibility of 30% according to the results of the survey ENSUSALUD 2014; assuming a confidence level of 95%. Since our study was a secondary analysis, we calculate the statistical power and the result was 99%.

The sampling was probabilistic, stratified, for each one of the 25 regions of Peru, and two-staged. Drugstores and pharmacies were considered as primary sampling units, while users who went to such establishments were the secondary sampling units.

### Eligibility criteria

The fourth questionnaire of ENSUSALUD included people who went and bought a medicine for themselves, their kid(s) or partner in a pharmacy or drugstore close to a health care establishment. For this study, we only included people who have bought medicine for themselves, and this was specified in the question (c4pa): Who did you buy medicine(s) for? Marking the alternative: “for myself”, so the self-medication definition of the WHO was met ^2^.

We excluded from the analysis the adults that bought medicines for their kid(s) or partner, who has not been able to go to the pharmacy and drugstore, since it was not possible to obtain the sociodemographic data and variables of interest of those people. A total of 1,226 (31.7%) people were excluded since they did not buy medicines for themselves (for their kid or partner). In addition, 55 participants were excluded for being under 18 years old. Finally, a total of 2,582 people were included in the study and they represented an expanded population of 2,057,592 people ( Figure 1). Despite the exclusion of 1,281 (33.2%) participants, the number of participants in the strata of the variables studied did not generated statistical differences.

### Variables and measurements

***Response variable.*** We used the question (c4p12): “These medicines, did you buy them with prescription?” to define the response variable. The answers were divided into three options: yes, and showed prescription=1; yes, and did not show prescription =2; no=3. Based on these categories, we created a dichotomous variable called self-medication (yes=1 and no=0), the self-medication category included those participants who bought medicines without doctor’s prescription and those who did not show the prescription when they were surveyed. Additionally, we categorized those participants who had self-medicated in two strata according to the type of medicine they bought (c4p11_1e): RSM (with OTC medicines) and ISM (without using OTC medicines). On the other hand, the non self-medication (NSM) category was composed of participants who bought medicines and showed doctor’s prescription when they were surveyed. Consequently, three final categories were generated (NSM=0; RSM=1; ISM=2).

***Exposure variables.***

Demographic, social and cultural factors

We included the following variables: sex (c4p3), age (c4p1), language (c4p5), education level (c4p4), current occupation (c4p6), guidance or help for self-medication (c4p21) and geographic region of residency (dominiog).

Factors associated with the Health System

We included the following variables: health insurance affiliation (c4p7), type of health insurance (c4p8) and the request of prescription by the pharmacist when buying the medicine (c4p19).

### Ethical considerations

ENSUSALUD 2015 is publicly accessible: http://portal.susalud.gob.pe/blog/base-de-datos-2015/. We downloaded the database without identifiers; thus, the confidentiality of the information given by the participants was guaranteed. Data collection was carried out after the verbal consent of the participants, it did not involve the biological sampling and was conducted for the management of health services nationwide.

### Statistical analysis

The database was downloaded from SUSALUD’s website in compatible format with the statistical package STATA ® v14.0 (Stata Corporation, College Station, Texas, USA). The database was programmed for a complex sample analysis, the regions of Peru were considered as strata; and drugstores, pharmacies and their users were considered as sampling units. The STATA module “complex survey data” (svy) was used.

The categorical variables were shown as absolute frequencies and as weighted proportions by the complex sampling, with their respective 95% confidence intervals (95%CI). Weighted proportions were calculated which allowed a comparison between the variable’s categories included in the analysis. In this way, we evaluated the association between variables, RSM and ISM practice through Pearson’s chi-squared test corrected for design purposes.

To evaluate the factors associated with RSM and ISM, multinomial logistic regression models were conducted (crude and adjusted) and the complex sampling of the study was considered (svy) ^22^. NSM was considered as the reference category.

The variables that showed statistically significant association (p<0.05) in the bivariate analysis, were included in the multinomial regression model. Possible collinearity relationships among variables was evaluated to obtain an adequate statistical consistency in the adjusted model. We developed a variable based on health insurance affiliation in participants (c4p7) and the type of health insurance (c4p8). This variable was evaluated in the multinomial regression models (crude and adjusted). The measure of association reported was the relative prevalence ratio (RPR), with their respective 95%CI. Moreover, we elaborated a second multivariate model that included variables whose association with self-medication has been described in the literature. However, this was similar to the first model prepared.

## Results

### General description of the population

We found that 57.4% participants were women, the average age was 41.4, 96.7% of the respondents spoke Spanish and only 25.3% of the participants had university education. Likewise, 69.4% of the respondents were affiliated to a health insurance, of which more than half were covered by the Comprehensive Health Insurance (SIS, by its Spanish initials) (52.8%) and EsSalud (40.0%).

The prevalence of NSM, RSM, and ISM was 25.2%, 23.8%, and 51.0% respectively. Only 27.7% of the participants were asked for their prescription by the pharmacist when buying the medicine. Furthermore, 54.6% of the participants received guidance or help by the drugstore or pharmacy personnel to self-medicate, and 13.1% of the participants resided in Lima ( Table 1).

**Table 1.** General characteristics of drugstores and pharmacies users, ENSUSALUD 2015 (N=2,057,592; n=2,582).

Characteristics	Absolute frequency of users surveyed	Weighted proportion of each category *	
	N	%	(95%CI)
**Gender**			
Female	1,481	57.4	(55.2-59.5)
Male	1,101	42.6	(40.5-44.8)
**Age**			
Average (95%CI)	41.4 (40.3-42.4)		
18 to 39	1,350	52.3	(49.2-55.4)
40 to 59	808	31.3	(29.1-33.6)
60 and older	424	16.4	(14.4-18.7)
**Language**			
Spanish	2,498	96.7	(94.7-98.0)
Quechua/Other	84	3.3	(2.0-5.3)
**Education level**			
University education ^§^	653	25.3	(22.2-28.7)
Non-university higher education ^§^	534	20.7	(18.6-22.9)
High school ^§^	974	37.8	(34.8-40.8)
Complete elementary education or below	419	16.2	(14.0-18.8)
**Current occupation**			
Dependent employee	659	25.5	(23.1-28.1)
Independent worker	958	37.1	(34.4-40.0)
Student	206	8.0	(6.8-9.4)
Housewife	591	22.9	(20.6-25.3)
Unemployed	109	4.2	(3.0-5.9)
Other	59	2.3	(1.6-3.4)
**Health insurance**			
Yes	1,792	69.4	(66.1-72.5)
No	790	30.6	(27.5-33.9)
**Type of health insurance** ^&^			
Comprehensive Health Insurance (SIS)	947	52.8	(47.2-58.5)
Social Security System (EsSalud)	717	40.0	(35.0-45.3)
Health Promoting Entities (EPS)	25	1.4	(0.8-2.3)
Health Insurance from Private Companies	30	1.7	(1.1-2.5)
Health Insurance from Private Clinics	13	0.7	(0.2-2.2)
College student health insurance	12	0.7	(0.3-1.4)
FF.AA.PP. Insurance	47	2.6	(1.8-3.8)
Other	1	0.1	(0.01-0.4)
**Self-medication**			
No	651	25.2	(21.7-29.1)
Responsible	614	23.8	(21.5-26.2)
Irresponsible	1,317	51.0	(47.8-54.2)
**Request of prescription by the pharmacist when** **medicine was sold**			
Yes	714	27,7	(23,9-31,7)
No	1,868	72.3	(68,3-76,1)
**Guidance or help for self-medication**			
Not applicable/not needed/other	370	14.3	(11.6-17.5)
Pharmacists	1,410	54.6	(49.6-59.5)
Radio/Newspapers or Magazines/Television	610	23.6	(19.8-27.9)
Internet	192	7.4	(5.9-9.4)
**Geographic region of residency**			
Metropolitan Lima	338	13.1	(8.5-19.6)
Other areas of Coast region	727	28.2	(21.2-36.3)
Highlands	1,070	41.4	(33.1-50.2)
Jungle	447	17.3	(11.9-24.5)

* Weight proportions and design effect of complex survey sampling were included.

^§^ Refers to complete or incomplete university, non-university higher education, or high school education.

^&^ Refers only to users who had health insurance.

### Description of drugs purchased by participants

When analyzing the total number of self-medicated users, it was found that the most commonly purchased medicine were non-steroidal anti-inflammatory drugs (NSAIDs) (24.4%), followed by antibiotics (16.5%) and analgesics/antipyretics/corticoids (16.4%). Also, the types of drugs most commonly purchased by participants who irresponsibly self-medicated were: NSAIDs (24.0%), antibiotics (22.6%) and gastrointestinal drugs (15.3%) ( Table 2).

**Table 2.** Types of medicine purchased by users who self-medicated (N=1,538,811; n=1,931), self-medicated irresponsibly (N=1,049,515; n=1317) and did not self-medicate (N=518,781; n=651).

Type of medicine purchased by participants	Self-medication N (%) *	Irresponsible self-medication N (%)	Non self- medication N (%)
Antibiotics	319 (16.5)	298 (22.6)	170 (26.1)
NSAIDs	471 (24.4)	316 (24.0)	109 (16.7)
Gastrointestinal	241 (12.5)	201 (15.3)	62 (9.5)
Analgesics/Antipyretics/Corticoids	317 (16.4)	144 (11.0)	58 (8.9)
Antihistamines/Respiratory pathologies	231 (12.0)	99 (7.5)	42 (6.5)
Nutritional supplement	104 (5.4)	53 (4.0)	46 (7.1)
Cardiac pathologies	89 (4.6)	57 (4.3)	51 (7.8)
Antiparasitic/Antiviral/Antimycotic	64 (3.3)	55 (4.2)	21 (3.2)
Metabolic disorders	41 (2.1)	41 (3.1)	37 (5.7)
Neurological pathologies	29 (1.5)	28 (2.1)	33 (5.1)
Other	25 (1.3)	25 (1.9)	22 (3.4)

* Includes irresponsible and responsible self-medication.

After evaluating the 651 participants who did not self-medicate, it was found that the main drugs purchased by this group were antibiotics (26.1%), NSAIDs (16.7%) and gastrointestinal drugs (9.5%) ( Table 2).

### Bivariate analysis

The absolute number of users per category of the variables studied was shown, in addition to the RSM and ISM percentages per each category. The corresponding weighting was considered.

No significant association was found between the practice of self-medication and language. However, there was statistically significant association with sex, age, education level, current occupation, having a health insurance, health insurance type, the request for prescription by the pharmacist when buying the medicine, guidance or help for self-medication, and region of residence ( Table 3).

**Table 3.** Percentage of responsible and irresponsible self-medication among users of drugstores and pharmacies of ENSUSALUD 2015 (N=2,057,592; n=2,582).

Characteristics	Absolute frequency of users per category	Weighted proportion of non self- medication according to each category *	Weighted proportion of responsible self- medication according to each category *	Weighted proportion of irresponsible self- medication according to each category *	
	N	%	%	%	p-value ^†^
**Gender**					
Female	1,481	28.0	23.4	48.6	0.001
Male	1,101	21.5	24.3	54.2	
**Age**					
18 to 39	1,350	20.1	28.1	51.8	<0.001
40 to 59	808	29.5	20.4	50.1	
60 and older	424	33.5	16.3	50.2	
**Language**					
Spanish	2,498	25.2	23.7	51.1	0.754
Quechua/Other	84	25.0	27.4	47.6	
**Education level**					
University education ^§^	653	23.0	26.5	50.5	0.044
Non-university higher education ^§^	534	22.4	24.0	53.6	
High school ^§^	974	25.1	22.9	52.0	
Complete elementary education or below	419	32.3	21.2	46.5	
**Current occupation**					
Dependent employee	659	23.6	25.3	51.1	<0.001
Independent worker	958	25.0	23.6	51.4	
Student	206	14.1	36.4	49.5	
Housewife	591	30.8	19.6	49.6	
Unemployed	109	28.4	20.2	51.4	
Other	59	23.7	13.6	62.7	
**Type of medical insurance**					
No	790	17.3	25.7	57.0	<0.001
Comprehensive Health Insurance (SIS)	947	30.4	22.9	46.7	
Social Security (EsSalud and EPS)	742	26.6	22.9	50.5	
Other **	103	28.2	23.3	48.5	
**Request of prescription by the** **pharmacist when medicine was** **sold**					
Yes	714	70.6	6.0	23.4	<0.001
No	1,868	7.8	30.6	61.6	
**Guidance or help for self-** **medication**					
Not applicable/not needed/other	370	38.1	18.4	43.5	<0.001
Pharmacists	1,410	25.5	20.7	53.8	
Radio/Newspapers or Magazines/Television	610	18.7	34.6	46.7	
Internet	192	18.7	22.4	58.9	
**Geographic region of residency**					
Metropolitan Lima	338	29.3	19.8	50.9	0.012
Other areas of Coast region	727	28.3	18.3	53.4	
Highlands	1,070	19.9	30.6	49.5	
Jungle	447	29.7	19.5	50.8	

* Weight proportions and design effect of complex survey sampling were included.

** It included the following categories: Health Insurance from Private Companies, Health Insurance from Private Clinics, College student health insurance, FF.AA.PP. Insurance.

^§^ Refers to complete or incomplete university, non-university higher education, or high school education.

† It refers to the statistical significance obtained from the comparison of proportions between categories of the variable considering the complex survey sampling.

### Multinomial logistic regression analysis

In the crude analysis, there was a greater RSM and ISM frequency in relation to male gender, current occupation (being a student) and not having a health insurance. Living in the Highlands region was associated with a higher frequency of RSM. Likewise, there was less frequency of RSM and ISM in relation to age (40 to 59 years and 60 to older), education level (complete elementary education or below) and current occupation (housewife) ( Table 4).

**Table 4.** Factors associated with responsible and irresponsible self-medication among users of drugstores and pharmacies, ENSUSALUD 2015 (N=2,057,592; n=2,582).

	Responsible self-medication						Irresponsible self-medication					
Characteristics	Crude Model *			Adjusted Model *			Crude Model *			Adjusted Model *		
	RPR	(95%CI)	p-value	RPR	(95%CI)	P-value	RPR	(95%CI)	p-value	RPR	(95%CI)	P-value
**Gender**												
Female	Reference			Reference			Reference			Reference		
Male	1.34	(1.06-1.70)	0.015	1.35	(1.06-1.72)	0.016	1.45	(1.19-1.76)	<0.001	1.41	(1.16-1.72)	0.001
**Age**												
18 to 39	Reference			Reference			Reference			Reference		
40 to 59	0.49	(0.37-0.66)	<0.001	0.53	(0.39-0.72)	<0.001	0.66	(0.51-0.85)	0.001	0.68	(0.53-0.88)	0.003
60 and older	0.35	(0.24-0.50)	<0.001	0.39	(0.25-0.59)	<0.001	0.58	(0.43-0.79)	0.001	0.65	(0.48-0.88)	0.005
**Education level**												
University education ^§^	Reference			Reference			Reference			Reference		
Non-university higher education ^§^	0.92	(0.66-1.30)	0.654	1.01	(0.70-1.44)	0.968	1.08	(0.79-1.49)	0.625	1.16	(0.84-1.60)	0.377
High school ^§^	0.79	(0.54-1.15)	0.213	1.04	(0.72-1.51)	0.836	0.94	(0.70-1.25)	0.668	1.12	(0.84-1.49)	0.444
Complete elementary education or below	0.57	(0.37-0.89)	0.014	0.94	(0.60-1.47)	0.770	0.66	(0.45-0.96)	0.028	0.92	(0.64-1.34)	0.674
**Type of Medical** **Insurance**												
Comprehensive Health Insurance (SIS)	Reference			Reference			Reference			Reference		
No	1.97	(1.35-2.87)	0.001	1.89	(1.31-2.71)	0.001	2.14	(1.52-3.01)	<0.001	2.03	(1.46-2.83)	<0.001
Social Security (EsSalud and EPS)	1.15	(0.75-1.74)	0.524	1.34	(0.90-2.00)	0.150	1.24	(0.85-1.81)	0.266	1.29	(0.88-1.88)	0.186
Other **	1.10	(0.58-2.08)	0.772	1.13	(0.60-2.14)	0.701	1.12	(0.66-1.92)	0.670	1.05	(0.61-1.81)	0.858
**Geographic region** **of residency**												
Metropolitan Lima	Reference			Reference			Reference			Reference		
Other areas of Coast region	0.95	(0.51-1.78)	0.882	1.02	(0.56-1.86)	0.937	1.08	(0.59-1.99)	0.793	1.14	(0.63-2.04)	0.665
Highlands	2.27	(1.22-4.23)	0.010	2.27	(1.23-4.21)	0.009	1.43	(0.79-2.61)	0.239	1.48	(0.82-2.70)	0.195
Jungle	0.97	(0.44-2.11)	0.932	0.98	(0.45-2.12)	0.962	0.98	(0.48-2.01)	0.961	1.04	(0.52-2.09)	0.914
**Current** **Occupation**				Not included ^§§^						Not included ^§§^		
Dependent employee	Reference						Reference					
Independent worker	0.87	(0.62-1.24)	0.448				0.94	(0.68-1.31)	0.724			
Student	2.40	(1.42-4.07)	0.001				1.62	(1.00-2.61)	0.049			
Housewife	0.59	0.42-0.83)	0.002				0.74	(0.56-0.98)	0.037			
Unemployed	0.66	(0.35-1.25)	0.200				0.83	(0.49-1.41)	0.489			
Other	0.53	(0.20-1.41)	0.201				1.22	(0.60-2.48)	0.590			

* A multinomial logistic regression model was performed considering the weighted proportions and design effect of the complex survey sampling.

** It included the following categories: Health Insurance from Private Companies, Health Insurance from Private Clinics, College student health insurance, FF.AA.PP. Insurance.

^§^ Refers to complete or incomplete university, non-university higher education, or high school education.

^§§^ Not included in the adjusted model due to collinearity with gender and type of medical insurance.

The factors associated with RSM in the adjusted analysis were male gender (RPR: 1.35; 95%CI: 1.06-1.72), not having health insurance (RPR: 1.89; 95%CI: 1.31-2.71) and living in the Highlands region (RPR: 2.27; 95%CI: 1.23-4.21). On the other hand, the only factors that remained associated with a lower frequency of RSM were being between 40 and 59 years old (RPR: 0.53; 95%IC: 0.39-0.72) and being 60 or older (RPR: 0.39; 95%IC: 0.25-0.59) ( Table 4).

The factors associated with a higher frequency of ISM in the adjusted analysis were male gender (RPR: 1.41; 95%CI: 1.16-1.72) and not having health insurance (RPR: 2.03; 95%CI: 1.46-2.83). Furthermore, the factors that remained associated with a lower frequency of ISM were being between 40 and 59 years old (RPR: 0.68; 95%IC: 0.53-0.88) and being 60 or older (RPR: 0.65; 95%IC: 0.48-0.88) ( Table 4).

## Discussion

This study found that three-quarters of the participants self-medicated and one out of two of the total population practiced ISM. Also, two out of three participants who irresponsibly self-medicated were not asked for the corresponding prescription when purchasing the medicine they wanted. The factors associated with increased RSM and ISM practice were male gender and not having health insurance. In addition, living in the Highlands was associated with RSM. On the other hand, being 40 to 59 years old or 60 to older were associated with a lower frequency of RSM and ISM.

Our study showed that the prevalence of self-medication was 74.8%, which was higher than that reported by Faria-Domingues *et al.* ^23^ in a systematic review that aimed to assess the prevalence of self-medication in adult population from Brazil. This review found that one-third of the population self-medicates. Likewise, the prevalence in our study was higher than that found by Jerez-Roig *et al*. ^24^ in a systematic review that included 28 studies predominantly from Brazil and the United States. Such review showed that the prevalence of self-medication in individuals aged 60 or older was on average 38%, and ranged from 4 to 87%. In our study, the participants were over 18 years old, this may explain the above-average prevalence mentioned in the systematic review of Faria-Domingues *et al*. ^23^.

Evidence from previous studies shows that self-medication frequency is higher in low and middle-income countries than in developed countries ^20^. Variable prevalence rates have been reported in Latin America, Africa and Asia (27%-86.4%) ^4, 5, 9– 13, 25^. However, percentages ranging from 8% to 14% ^1^ are reported in developed countries (United Kingdom, Italy, Switzerland, Belgium, Germany, France, United States of America and the United Kingdom). This is due to the fact that in these countries there is an adequate supervision when supplying OTC drugs. Therefore, most of these prevalence’s correspond to the purchase of OTC medicines. It has also been reported that certain types of drugs such as antibiotics and NSAIDs are available as OTC drugs, causing adverse reactions due to misuse ^1, 16, 19, 20^.

The most requested drugs by the participants were NSAIDs, antibiotics and analgesics/antipyretics/corticoids. A previous study in Peru found that NSAIDs were also the most acquired drugs (30%) ^14^. On the other hand, in a study carried out in Colombia, the most commonly used drugs were analgesics/antipyretics (44.3%), NSAIDs (36.4%) and antihistamines (8.5%), which goes according to our findings ^10^. These findings are similar because analgesics/antipyretics/corticoids, NSAIDs, antihistamines, and antibiotics are the most frequently used drugs described in other studies ^7, 9, 26– 31^ and are used to treat common symptoms that people do not consider sufficient reason to see a doctor; therefore, they tend to self-medicate.

Our study showed that the male gender reported a higher self-medication frequency. However, studies such as those of Jerez-Roig *et al*. ^24^ and Lukovic *et al.* ^32^ reported that the female gender is associated with a higher prevalence of self-medication practice. Our findings are further supported by the study of Quédraogo *et al.* ^33^, who described that the self-medication practice was found to be associated mostly with the male gender in rheumatic diseases. However, this study was only carried out in an urban area; therefore, the results could vary or not be extrapolated at rural or national level, as is the case of our research.

In Peru, a study conducted in a district of Metropolitan Lima by Hermoza-Moquillaza *et al.* ^14^ described that self-medication percentage was higher in the male gender. However, this study did not carry out regression models with multiple variables, which would give our study an innovative character because it is a population-based study and includes a multivariate analysis. On the other hand, findings in Peru regarding a lower self-medication prevalence in females can probably be explained by the sexist nature of its society ^34^. We consider that, in sexist societies, the family structure implies that women are relegated to housekeeping and taking care of children, which, together with the repression from their partner, would reduce their probability to self-medicate and even access to health services. This association could also be explained because men would spend most of their time at work and would not have enough time to go to a health center, having less access to health services ^35, 36^, so they would resort to the practice of self-medication. Besides, the high prevalence of NSM in women and older adults could be explained due to their poor health status, which would predispose them to use frequently health services and receive a medical prescription. Furthermore, older adults need to acquire several drugs for the best management of their comorbidities ^37^.

A higher self-medication prevalence was found in participants without health insurance compared to those with SIS. Not having health insurance prevents patient from accessing health services, with the option of self-medicate in drugstores and pharmacies. In this context, some studies have associated the difficulty in obtaining a medical appointment with the self-medication practice, as well as an adverse financial situation ^38– 41^. However, in some health systems, having a health insurance does not guarantee a lower self-medication prevalence. Thus, no statistically significant differences were found between people who have SIS or EsSalud and the practice of RSM or ISM. This situation could be explained by the fact that those with SIS can easily make an appointment as an outpatient, but they would not have an adequate access to medicines ^21^. In contrast, the situation with social security is the opposite, people with this insurance can benefit from the adequate supply of medicines under this contributory system. However, making an appointment as an outpatient would represent a more problematic situation compared to people with SIS ^42^. Both situations would eventually lead to RSM or ISM. A similar case occurs in China, where long waiting times (more than half a day), and an expensive medical care, would lead to a high self-medication prevalence of antibiotics in college students ^43^. Similarly, in Saudi Arabia, there was a positive association between the difficult access to health services and the self-medication practice of patients in primary care centers ^44^.

Peru has a fragmented health system, which is divided into two sectors: public and private ^45, 46^. The public sector is also divided into subsidized or indirect contributory system and direct contributory or social security system. In the public sector, the government provides medical services (SIS) to the population living in poverty through the MINSA-GR establishments. The social security system is intended for citizens with formal employment. It has two subsystems: EsSalud and the private health care providers. Furthermore, the FF.AA.PP have their own health subsystem. Finally, the private sector is divided into the for-profit system (private insurance companies, private clinics) and non-profit system (NGOs) ^45, 46^. The process of universal health insurance in Peru has begun since 2009 and seeks to ensure that more Peruvians have medical insurance based on an essential plan. However, this process is still being implemented and many citizens do not have health insurance yet ^47^. This leads to a lack of access to medical services and a high prevalence of ISM in Peru ^48, 49^.

We found an association between the lack of request for prescription by the pharmacist when purchasing the medicine and self-medication in the bivariate analysis. This situation is evidenced by the inadequate distribution of prescription medicines, as is the case with the OTC sale of antibiotics in Peru, despite the current regulations ^14, 50^. This situation also occurs in other Latin-American countries such as Chile and Colombia, where there are regulations to prevent the free distribution of antibiotics; however, the results are not evidenced over the years ^26, 51^. We also observed a small percentage of participants (6%) who were asked for a prescription despite purchasing an OTC medicine. This would reflect a professional malpractice by pharmacists in Peru.

This study has limitations: 1) since it is a secondary analysis of a survey designed to assess user’s satisfaction of health services, it was not necessarily conducted to answer our research question; however, the questionnaire has been designed and validated by the INEI staff; 2) the cross-sectional design of this study does not allow us to establish a causal relationship among the factors associated with self-medication. However, it allows us to find the association and identify the markers that could be used by healthcare managers to carry out future public health interventions ^52^.

In conclusion, there is a high self-medication frequency in Peru, mostly with medicines that are not authorized for OTC sale. It is important to carry out public health interventions in order to reduce the ISM frequency in Peru. There is also a need for educational reforms aimed at raising awareness of the consequences of this irresponsible practice. Similarly, respective measures should be taken to improve the coverage of universal health insurance, thus preventing people from resorting to ISM due to lack of access to medical services. Finally, efforts should be made to integrate druggists and pharmacists into regulatory entities in order to control OTC and uncontrolled sales of prescription drugs. Self-medication could represent a quick and economical solution for users, but it must be practiced within a responsible context.

## Data availability

### Underlying data

Data associated with this study is available at National Superintendency of Health ( *Superintendencia Nacional de Salud,* SUSALUD) website: http://portal.susalud.gob.pe/blog/base-de-datos-2015/

### Extended data

Questionnaire analyzed is available online: http://portal.susalud.gob.pe/wp-content/uploads/archivo/encuesta-sat-nac/2015/Cuestionario-4-DIRIGIDA-A-USUARIOS-DE-FARMACIAS-BOTICAS.pdf
