# Supplementary material for: Factors associated with self-medication in users of drugstores and pharmacies in Peru: an analysis of the National Survey on User Satisfaction of Health Services, ENSUSALUD 2015
Source: F1000Res. 2020 Jan 24;8:23. Originally published 2019 Jan 7. [Version 2] doi: 10.12688/f1000research.17578.2 (PMC7001751; doi:10.12688/f1000research.17578.2)
Supplement: Peer review history

# Peer review: Reviewer response for version 2

**DOI:** 10.5256/f1000research.24287.r59062

**Published:** 2020-02-03

**Authors:** Marcus Tolentino Silva

**Affiliations:** 1 University of Sorocaba, Sorocaba, Brazil

I have no further comments to make.

I confirm that I have read this submission and believe that I have an appropriate level of expertise to confirm that it is of an acceptable scientific standard.

# Peer review: Reviewer response for version 1

**DOI:** 10.5256/f1000research.19222.r42721

**Published:** 2019-05-28

**Authors:** Cristian Díaz-Vélez

**Affiliations:** 1 Oficina de Inteligencia Sanitaria, Hospital Nacional Almanzor Aguinaga Asenjo EsSalud, Chiclayo, Peru

1. The sample calculation mentions the use of 30% satisfaction, but it is not a used variable.
2. The first paragraph of the discussion should go into the results section.
3. It shows results of types of drugs most used in self-medication, but in the discussion this is not mentioned. See existing studies published in Peru (Lima and Lambayeque).
4. Another limitation of the study not mentioned, is the effect of the pharmacies in the dispensing of medicines, when changing or indicating drugs. See Study: Alterations in drug dispensation by private sector pharmacies in the district of Chiclayo ^1^.

I confirm that I have read this submission and believe that I have an appropriate level of expertise to confirm that it is of an acceptable scientific standard.

## References

[1] : [Alterations in drug dispensation by private sector pharmacies in the district of Chiclayo]. *Rev cuerpo méd HNAAA*.2012;5(1) :26-29 Reference source

# Response

**Published:** 2020-01-15

**Authors:** Diego Urrunaga-Pastor

**Affiliations:** USIL, Peru

We have read the reviewers commentaries and want to clarify certain points in this new version:

 Dear Dr. Cristian Díaz-Vélez:

 1. The sample calculation mentions the use of 30% satisfaction, but it is not a used variable.

 Answer: Thank you for the observation, ENSUSALUD was developed to evaluate health user satisfaction, therefore the INEI did not perform a sample size calculation based on self-medication prevalence.

 2. The first paragraph of the discussion should go into the results section.

 Answer: Thank you for the suggestion, however, we consider keeping the paragraph as a summary of our main findings.

 3. It shows results of types of drugs most used in self-medication, but in the discussion, this is not mentioned. See existing studies published in Peru (Lima and Lambayeque).

 Answer: Thank you for the commentary, in the 4th paragraph of the discussion section we have cited international and national studies to mention these results.

 4. Another limitation of the study not mentioned is the effect of the pharmacies in the dispensing of medicines when changing or indicating drugs. See Study: Alterations in drug dispensation by private sector pharmacies in the district of Chiclayo1.

 Answer: Thank you for the suggestion, however, it is not part of the objectives of our study and we did not have an instrument to measure this variable.

# Peer review: Reviewer response for version 1

**DOI:** 10.5256/f1000research.19222.r42722

**Published:** 2019-01-23

**Authors:** Marcus Tolentino Silva

**Affiliations:** 1 University of Sorocaba, Sorocaba, Brazil

This is a national survey in Peru performed in drugstores and pharmacies in 2015. The authors stratified their respondents in: (i) non self-medication (NSM, purchased based a prescription); (ii) responsible self-medication (RSM, paid an OTC); or (iii) irresponsible self-medication (IRM, acquire a prescription drug without prescription). The results reveal that RSM and IRM are more frequent in males and without health insurance, and more uncommon in >39 years old.

 Some suggestions for improvement:

1. In Table 1, please stratify the characteristics of participants by the three response variables (NSM, RSM, IRM) and exclude the Table 2 (male data are missing). Change the Figure 2 to table for better visualization.
2. I believe that the results are influenced by the NSM group. This population had a prescription, which is, visited more health services and probably had poor health status. Such characteristics are attributable to women and elders. The poor health status of the NSM group induced their best behavior. Sexism is important, but I think that it is not sufficient to explain worse male health behavior. Please review these aspects in the discussion.

I confirm that I have read this submission and believe that I have an appropriate level of expertise to confirm that it is of an acceptable scientific standard, however I have significant reservations, as outlined above.

# Response

**Published:** 2020-01-15

**Authors:** Diego Urrunaga-Pastor

**Affiliations:** USIL, Peru

We have read the reviewers commentaries and want to clarify certain points in this new version:

 Dear Dr. Marcus Tolentino-Silva:

 1. In Table 1, please stratify the characteristics of participants by the three response variables (NSM, RSM, IRM)

 Answer: Thank you very much for the commentary, however, we consider important to keep table 1 with the descriptive analysis and to present the 3 strata of our main variable (NSM, RSM, ISM) in table 2.

 2. Exclude the Table 2 (male data are missing)

 Answer: Thank you for the observation, we have added the missing data in table 3. In addition, we have presented the bivariate analysis based on the 3 strata of the main variable.

 3. Change the Figure 2 to table for better visualization.

 Answer: We appreciate the comment. We have removed figure 2 and replaced it by a table (table 2) for better visualization.

 4. I believe that the results are influenced by the NSM group. This population had a prescription, which is, visited more health services and probably had poor health status. Such characteristics are attributable to women and elders. The poor health status of the NSM group induced their best behavior

 Answer: Thank you for the commentary, we have reviewed the literature, expanding this discussion section with this information.

 5. Sexism is important, but I think that it is not sufficient to explain worse male health behavior. Please review these aspects in the discussion.

 Answer: Thank you for the commentary, we did a review of the literature regarding this topic, expanding the information presented in the discussion.
